# Neighborhood health effects on the physical health of the elderly: Evidence from the CHRLS 2018^☆^

**DOI:** 10.1016/j.ssmph.2022.101265

**Published:** 2022-10-11

**Authors:** Hu Yangming, Gong Rengui, Zeng Long

**Affiliations:** College of Public Administration and Law, Hunan Agricultural University, Changsha, China

**Keywords:** Elderly people, Physical health, Neighborhood health effects, Mechanism of function

## Abstract

**Background:**

There are more than 26 million elderly people in China, and due to the Health China strategy proposed in 2020, “Elderly Health” has become an important topic of concern for all sectors of society. Neighborhoods provide important social relationships. However, Chinese researchers have not extensively explored the impact of these relationships on the physical health of the elderly.

**Methods:**

Based on the data from the 2018 China Health and Retirement Longitudinal Study (CHARLS), we constructed a comprehensive research framework integrating ordinary least square (OLS) regression, heterogeneity analysis, IV-2SLS, robustness testing, and Karlson–Holm–Breen (KHB) mediating effect analysis, which can be used to thoroughly examine neighborhood health effects (NHEs) in relation to the physical health of the elderly.

**Results:**

The OLS results showed that the NHEs (B = 0.4689, p < 0.01) had a positive influence on the physical health of the elderly, and were lower than the NHEs estimated by IV-2SLS (B = 0.5018, p < 0.01). The mediating effects of social networks and social relationships were analyzed using KHB, and both the total (B = 0.6056, p < 0.01) and indirect (B = 0.0800, p < 0.01) effects on neighborhood health were significant, with the total effect being 10 times larger than the direct effect and 13.24% of the total effect coming from the mediating variable.

**Conclusions:**

Firstly, the NHEs positively influence the physical health of elderly persons, but there are heterogeneous differences. Secondly, the IV-2SLS estimation results suggest that not controlling for endogeneity leads to underestimation of the role of the NHEs. Thirdly, using the county-level NHEs, self-rated health, and health changes to replace variables, and grouping by smokers (small sample) and never smoked (large sample), the influence of the NHEs on the physical health of the elderly is robust. Finally, social networks and social relationships are important transmission mechanisms of the NHEs when it comes to the physical health of the elderly.

## Highlights

• NHEs are robust and significant in the Chinese social background.
• Community (county) level NHEs are positive for the positive health of the elderly.
• NHEs have a positive impact on self-rated health and health changes in older adults.
• Endogenous issues would underestimate the role of NHEs.
•Social networks and social relationships are transmission mechanisms of the NHEs.

## 1 Introduction

Promoting a long and healthy life for the elderly is the basis for ensuring the quality of life of the elderly and maintaining their dignity. As social development and medical progress continue in China, the number of elderly people in the country is increasing and creating a societal imbalance. According to the China Population Census-2020,^1^ the current population of people aged 60 and over in mainland China is 264.02 million, accounting for 18.70% of the total population (National Bureau of Statistics of China, 2021). To respond to this aging trend and improve the health of the elderly, the Chinese government has built many squares and parks to promote group fitness exercises such as Tai Chi and square dancing. This public policy has been successful, and today the elderly are highly motivated to participate in group fitness exercises. Tai Chi and square dance have been shown to improve physical health and immune function in elderly persons, as well as reduce their feelings of loneliness and increase their life satisfaction (Su & Zhao, 2021). In fact, whether it is Tai Chi and square dance, or the fitness trails and cycling paths that are being actively promoted in many parts of China, scenes of families or neighbors exercising together are common. An old Chinese saying is that a distant relative is not as good as a near neighbor. Psychological and behavioral studies also show that individual behavior is not only influenced by individual factors and family, but also by group behavior (Ali & Masood, 2015; Ivory et al., 2015; Ross & Mirowsky, 2001).

Currently, social science research on the health of the elderly revolves around four main pathways. The first of these is the individual factors of the elderly. An individual elder's health is affected by personal history, basic characteristics, and behavioral habits. Mello systematically combed 182 articles to summarize factors associated with elder health, such as age, gender, education, income, number of diseases, self-assessed health, depressive symptoms, smoking, and alcohol consumption. (Mello et al., 2014). Feng et al. have studied changing trends in the health of elderly Chinese, and reported that it is influenced by factors such as pension, occupation before age 60, household registration, and more (Feng et al., 2019). E-health services have played an important role in improving health literacy and health-care support for the elderly. A research study showed that 81% of the elderly use the Internet to obtain information about health or illness (Bujnowska-Fedak & Mastalerz-Migas, 2015). Two articles published in top journals in China both came to the same conclusion: using the Internet can improve the self-rated health of the elderly, and makes a significant positive contribution to achieving active aging (Jin & Zhao, 2019; Zhao & Liu, 2020). The second main pathway is family factors. Family resources, family care, and household chores will affect the health of the elderly. The most significant component of family resources is family income, and some studies have shown that elderly people with low family income have poorer health (Hinata et al., 2021). Family orchards are one family resource that affects family economic income, and orchard work contributes to improved physical health as well as mental health (Madruga et al., 2021). At present, adult children are primarily responsible for home care of the elderly in China, but urban development has resulted in many adult children migrating away from their families. There is evidence that migration of adult children can impair the health of the elderly (Li et al., 2020). The third main pathway is social participation, which has a preventive impact on disease, especially in the elderly (Kanamori et al., 2014; Otsuka et al., 2018). Ma addressed reverse causality and heterogeneity through a lagged-variable (LV) approach and a random-effects (RE) model, and the study demonstrated that social engagement positively influenced health outcomes among middle-aged and elderly Chinese (Ma et al., 2020). A study in Sardinia, a “Blue Zone” which has one of the longest average human lifespans in the world, has shown that participation in leisure activities is beneficial in improving happiness and reducing depressive symptoms (Fastame et al., 2018). Finally, the social welfare system factor is the fourth main pathway. Social welfare systems such as medical insurance and pension insurance are considered to have positive effects on the health of the elderly. Medical insurance is an important factor in improving health care utilization, but different medical insurance programs have various effects on middle-aged and elderly person (Zhou et al., 2020). A review of long-term care insurance (LTCI) concludes that implementation of the LTCI system not only protects the health of caregivers and care recipients, but also reduces the financial burden on families (Chen & Xu, 2020).

But most studies view the health of individual elderly persons as isolated from that of the elderly group. Early public health and epidemiological researchers emphasized the need to consider not only individual characteristics but also characteristics of the group or background to which an individual belongs when studying the distribution of health and disease. Scholars refer to this phenomenon as “neighborhood health” (Kawachi & Berkman, 2009; Wilson, 1987), and there are two main lines of research. On the one hand, attention is paid to the neighborhood social environment, in terms of aspects such as socioeconomic status, social interaction, race, and crime levels (Liu & Wang, 2022; Sun et al., 2020; Zhang & Wu, 2017). On the other hand, attention is paid to the physical environment of the neighborhood, such as land-use patterns, transportation systems, medical service points, and fitness-facility layouts (Diez & Mair, 2010; Gustat et al., 2014; Liu et al., 2018). Research on NHEs in China is still in its infancy, and the mechanisms and results of neighborhood effects on residents' health are still unclear (Liu et al., 2018). From the perspective of traditional Chinese culture and Chinese behavioral habits, it is highly likely that there is a strong neighborhood effect on the individual health of the elderly. Although the traditional society of acquaintances has been transformed to a certain extent into a society of strangers, “the Pattern of Difference Sequence” has not completely failed, because Chinese society has been constructed on the basis of blood and geography since ancient times. (Fei et al., 1992). Chinese society is group-oriented and values daily communication and interaction between people. This lifestyle has led to Chinese people's thoughts and behaviors being easily influenced by other members of the group. With the rise of “Healthy China”, more and more elderly people are participating in fitness exercises and leisure activities in groups. In summary, studying the relationships between neighborhood health and the individual health of the elderly, and clarifying the mechanism of the neighborhood effect, provides a new perspective for understanding the increasing construction of community leisure and sports facilities and smart community adaptation in China.

## 2 Materials and methods

### 2.1 Study sample

We used data from the China Health and Retirement Longitudinal Study (CHARLS), a large-scale long-term tracking survey project implemented by Peking University to address the challenges of China's aging population. CHARLS 2018 surveyed 11,797 households and 20,284 residents in 28 provinces and 150 counties in China, and provides the most recent data currently available. During the fieldwork, each respondent who agreed to participate in the survey was asked to sign two copies of the informed consent form. Ethical approval for all the CHARLS waves was granted by the Institutional Review Board at 10.13039/501100007937Peking University. The IRB approval number for the main household survey, including anthropometrics, is IRB00001052-11015. CHARLS 2018 data cover basic social characteristics of the population, health status and functioning, health care and insurance, work and retirement, and family status, important data points for this study on the impact of neighborhood effects on the health of the elderly. We excluded missing data, made necessary calculations, and merged some of the data, so that the final data used in the study comprised 126 cities, 449 neighborhoods, and 9921 individuals. The statistical software we used was Stata/SE 17.0.

### 2.2 Variables

**Explained variable: Physical health.** The explained variable we focused on was physical health in the elderly. The CHARLS household questionnaire asked respondents whether they had difficulty with seven activities: running or jogging about 1 km; getting up from a chair after sitting for a long period; climbing several flights of stairs without resting; stooping, kneeling or crouching; reaching or extending their arms above shoulder level; lifting or carrying weights over 10 kg; and picking up a small coin from a table. There were four answers to choose from: “No, I don't have any difficulty”, “I have difficulty but can still do it”, “Yes, I have difficulty and need help”, and “I cannot do it”. To facilitate understanding and comparison, these four answers were respectively given 4, 3, 2, and 1 point in data processing, and then the scores for the seven responses were added up and divided by seven. The interval of physical health values for the elderly was [1,4], with 1 point indicating the worst health status and 4 points indicating the best.

**Key explanatory variable: Neighborhood health effects.** It was necessary to fully consider the geographical scope of the daily communication and interaction of the elderly. If the neighborhood range were set too large, neighborhood health effects would be minimal, and our study would be meaningless. Given that communication and interaction among different elderly people mainly occur within the community, this study focused on examining NHEs at the community level. Referring to Manski's social-interaction-effect model and Ling et al.'s approach (Ling et al., 2018; Oakes, 2004), we define the community NHEs as: the average health level of elderly people in community I except the elderly individual i. The specific calculation of the NHEs are shown in Eq. (1)：

(1) $$N_{i}=\left(H_{I}-H_{i}\right)/\left(n-1\right)$$

In Eq. (1), *N*_*i*_ represents the NHEs of the elderly individual i, $H_{I}$ represents the sum of the physical health levels of all elderly in community I, *H*_i_ represents the physical health level of elderly person *i*, and n represents the number of elderly people in community I.

**Instrumental variables: Fitness exercise and leisure activity of other elderly people.** Physical health of the elderly and NHEs may be endogenously influenced by mutual causality, and instrumental variable estimation (IV) is an effective method to solve the difficulty of identifying neighborhood effects. Fitness exercise and leisure activity have effects on the health of specific elderly people, but may have smaller effects on the health of others, which may well satisfy the correlation and exogeneity conditions of the instrumental variables. Based on the approach of Dr. An, this paper uses the average fitness exercise and average leisure activity of other elderly people in the community as the instrumental variables for NHEs (An, 2015).

**Mediating variables: Social networks and social relationships.** Variable definitions: whether or not an elderly person had been online in the past month was used as a proxy variable for social networks (Booth et al., 2018), and ln(cash gifts) in the past year were used as a proxy variable for social relationships (Subramanian et al., 2006).

**Control variables.** Referring to the published literature on studying NHEs (such as Subramanian et al. (Subramanian et al., 2006), Jennings et al.(Jennings et al., 2018), and Chaix et al. (Chaix et al., 2011)) we controlled for the following types of variables. One was individual basic status, including gender, age (60 years and above), marital status, education, and household registration. The second was economic status. The elderly person's income directly affects nutritional intake, quality of life, etc., thus affecting health. Therefore, total annual income (including subsidized income) was used as a proxy variable for economic status. The third variable was family care status. Currently, the main elder-care model in China is family care, and the elderly are mainly taken care of by sons. Therefore, we included the following three variables into the model: proportion of sons among all adult children, financial support from all adult children, and care time from all adult children. The fourth variable was public medical policy. Medical service is an important guarantee for the physical health and psychological health of the elderly, but considering that only 163 people (1.62% of the total sample) did not participate in the public health insurance plan, we included options for receiving paid family doctor services and medical service satisfaction.

The definitions of the above variables and descriptive statistics are shown in Table 1.

**Table 1:** Definition of variables and descriptive statistics.

Types	Variables	Value and meaning	Mean	SD	Min	Max
Explained variables	Physical health	Physical health	3.291	0.673	1	4
Key explanatory variable	NHEs	Neighborhood health effects (Calculated by Eq. (1))	3.383	0.17	2.397	3.888
Instrumental Variables	NFEs	Neighborhood fitness exercises (Calculated, like NHEs)	0.060	0.059	0	0.421
	NLAs	Neighborhood leisure activities (Calculated, like NHEs)	0.479	0.170	0	1
Mediating Variables	Online	Online within the past month = 1, otherwise = 0	0.06	0.238	0	1
	lnCG	ln(cash gifts) (logarithmic calculation)	3.219	3.63	0.095	13.305
Control variables	Gender	Male = 1, Female = 0	0.487	0.5	0	1
	Age	Age in 2018	69.186	7.091	60	118
	Marital status	With spouse = 1, otherwise = 0	0.758	0.428	0	1
	Education	No formal education = 1, Did not finish primary school = 2, Elementary school = 3, Middle school = 4, High school = 5, otherwise = 6	2.535	1.396	1	6
	Household registration	Agricultural = 1, otherwise = 0	0.694	0.461	0	1
	lnTPI	ln(total personal income)	7.163	3.014	0.095	13.306
	Proportion of sons	Proportion of sons among all adult children	0.542	0.296	0	1.1
	lnFSC	ln(Financial support from adult all children)	4.045	3.457	−1.609	10.309
	Care time from adult children	Care time from all adult children	2.134	3.015	0	12
	Family Doctor Services	Received paid family doctor services = 1, otherwise = 0	0.044	0.206	0	1
	Medical service satisfaction	Very dissatisfied = 1, somewhat dissatisfied = 2, neutral = 3, somewhat satisfied = 4, very satisfied = 5	3.334	1.12	1	5

### 2.3 Statistical analyses

**OLS model.** We were concerned with the impact of the NHEs on the health of the elderly and its functioning mechanism. Therefore, we introduced the NHEs into the model as the key explanatory variable during the model construction process. Since the explanatory variable “health of the elderly” was a continuous variable, the basic model used OLS regression, as shown in Eq. (2):

(2) $$Y_{i}=\alpha_{11}+\alpha_{12}{NHEs}_{i}+\beta_{11}C_{i}+\varepsilon_{1i}$$

In Eq. (2), *Y*_*i*_ and *NHEs*_*i*_ represent the physical health and neighborhood health effects of the elderly i, respectively, and *C*_*i*_ represents the control variables, including gender, age, marital status, education, household registration, lnTPI, proportion of sons, lnFSC, care time from adult children, family doctor services, and medical service satisfaction. The coefficient α_12_ was the focus of this study, reflecting the impact of the NHEs on the health of the elderly individual. $\alpha_{11}$ represents the constant, $\beta_{11}$ represents the coefficient of the control variable, and $\varepsilon_{1i}$ represents the random disturbance.

The OLS model adopts a step-by-step method of adding variables and is divided into five steps. Model (I) is the regression result with NHEs only. Model (II) adds individual basic status of the elderly individual (I). Model (Ⅲ) also adds the economic status of the elderly individual (Ⅱ). Model (Ⅳ) is the regression result of the model (Ⅲ) with the addition of three proxy control variables for family care. Model (V) adds two proxy control variables for public medical policy to model (IV). The OLS regression results showed that the estimated coefficients of the NHEs were always positive and significant at the 1% level, after controlling for individual basic status, economic status, family care status, and public medical policy, indicating that the physical health of the elderly was always positively influenced by the NHEs. The results for model (V) indicated that each 1-unit increase in neighborhood health contributed to a 0.489-unit increase in the health of elderly persons.

**IV-2SLS model.** Fitness exercises and leisure activities may have an impact on the health of specific older adults to some degree, but may have a smaller impact on the health of other older adults. So, Fitness exercises and leisure activities can satisfy the requirements conditions of correlation and exogeneity of instrumental variables. Therefore, we constructed two instrumental variables: NFEs (Neighborhood fitness exercises), NLAs (Neighborhood leisure activities), and used 2SLS to retest the relationship between NHEs and the physical health of older adults. Then, we used 2SLS to restudy the relationship between NHEs and the physical health of the elderly. The first step of 2SLS is to regress the instrumental variables (NFEs, NLAs) using NHEs to obtain the fitted values of NHEs*, with the following Eq. (3):

(3) $${NHEs}_{i}=\alpha_{211}+\alpha_{212}{NFEs}_{i}+\alpha_{213}{NLAs}_{i}+\beta_{211}C_{i}+\varepsilon_{21i}$$

(4) $$Y_{i}=\beta_{221}+\beta_{222}{NHEs}_{i}^{*}+\beta_{221}C_{i}+\varepsilon_{22i}$$

Then, the 2SLS second-stage regression estimation was completed using the fitted values of NHEs (Eq. (4)). $Y_{i}$ represents the physical health, $\alpha_{211}$ and $\beta_{221}$ represent the constants, $\alpha_{212}$ and $\alpha_{213}$ represent the instrumental variables' coefficients, $\beta_{222}$ represents the ${NHEs}^{*}$ coefficient, $\beta_{211}$ and $\beta_{221}$ represent the control variables' coefficients, $\varepsilon_{21i}$ and $\varepsilon_{22i}$ represent the random disturbances.

**KHB model.** The assumption was that if the elderly person was often online, they were more likely to access and share health information, and that therefore the Internet played a role in the NHEs by encouraging the elderly to adopt more healthy behaviors. We used the KHB mediating effect analysis approach to explore the mediating effects of social connection and social relationships, and then calculated the proportion of direct and indirect effects that could be explained by these factors. With the following equation:

(5) $$Y_{i}=\alpha_{311}+\alpha_{312}{NHEs}_{i}+\beta_{311}C_{i}+\gamma {M_{i}+\varepsilon }_{31i}$$

(6) $$Y_{i}=\alpha_{321}+\alpha_{322}{NHEs}_{i}+\beta_{321}C_{i}{+\varepsilon }_{32i}$$

(7) $$R=M-\left(\alpha_{331}++\beta_{331}C_{i}\right)$$

In Eq. (5), $Y_{i}$ represents the physical health, $\alpha_{311}$ represents the constant, $\alpha_{312}$ represent NHEs coefficient, $C_{i}$ represent the control variables, $\beta_{311}$ represents the coefficients of the control variables, M represents the mediating variable, γ represents the coefficient of the mediating variable, and $\varepsilon_{32i}$ represents the residual. Eq. (6) is the parsimonious model excluding the mediating variables, where $\alpha_{321}$, $\alpha_{322}$ and $\beta_{321}$ represent the constant, coefficient of NHEs, and coefficient of control variables, respectively. The idea of Eq. (7) is to extract information from M that is not contained in the NHEs. This is done by calculating the residuals R of the linear regression of M on NHEs, where $\alpha_{331}$ and $\beta_{331}$ are the estimated regression parameters of the linear regression. Replacing the mediating variables of Eq. (8) with R yields the following equation.

(8) $$Y_{i}=\tilde{\alpha_{311}}+\tilde{\alpha_{312}}{NHEs}_{i}+\tilde{\gamma }R+\tilde{\beta_{311}}C_{i}+\varepsilon_{41i}$$

In the next step, the difference between $\alpha_{322}$ and $\tilde{\alpha_{312}}$ is tested for significance, and if the difference is significant then the mediating effect holds. Specifically, on the OLS baseline model, we separately added the two mediating variables, social connection (“Online”) and social relationships (“cash gifts”), and then added them both simultaneously to build the full model.

## 3 Results

### 3.1 OLS regression analysis

The OLS regression results (Table 2) showed that the estimated coefficients of the NHEs were always positive and significant at the 1% level, after controlling for the basic characteristics of the elderly, their economic situation, their family care, and public health policy variables; this meant that the physical health of the elderly was consistently and positively influenced by the NHEs. The results for model (V) indicated that each 1-unit increase in neighborhood health contributed to a 0.489-unit increase in the health of the elderly. The results in column (5) of Table 2 show that gender (B = 0.2180, p < 0.01), marital status (B = 0.0351, p < 0.05), education (B = 0.0562, p < 0.01), lnTPI (B = 0.0204, p < 0.01), proportion of sons (B = 0.0487, p < 0.05), lnFSC (B = 0.0063, p < 0.01), care time from adult children (B = 0.0046, p < 0.05), and medical service satisfaction (B = 0.0497, p < 0.01), significantly and positively influenced the health level of the elderly person, while age (b = −0.0282,p < 0.01) significantly and negatively influenced their health level (Hinata et al., 2021). However, household registration and family doctor services had no significant effect on the physical health of the elderly person.

**Table 2:** Neighborhood health effects on the elderly (OLS).

Variables	（I）	（Ⅱ）	（Ⅲ）	（Ⅳ）	（V）
Neighborhood health effects	0.6055***	0.5143***	0.4757***	0.4683***	0.4689***
	(0.0395)	(0.0365)	(0.0365)	(0.0367)	(0.0365)
Gender		0.2200***	0.2130***	0.2133***	0.2180***
		(0.0132)	(0.0132)	(0.0131)	(0.0131)
Age		−0.0275***	−0.0283***	−0.0279***	−0.0282***
		(0.0010)	(0.0010)	(0.0010)	(0.0010)
Marital status		0.0342**	0.0322**	0.0351**	0.0351**
		(0.0152)	(0.0152)	(0.0152)	(0.0151)
Education		0.0601***	0.0526***	0.0521***	0.0562***
		(0.0050)	(0.0050)	(0.0050)	(0.0050)
Household registration		−0.0194	0.0146	0.0116	0.0032
		(0.0140)	(0.0144)	(0.0144)	(0.0143)
lnTPI			0.0206***	0.0208***	0.0204***
			(0.0022)	(0.0022)	(0.0022)
Proportion of sons				0.0483**	0.0487**
				(0.0195)	(0.0194)
lnFSC				0.0060***	0.0063***
				(0.0018)	(0.0018)
Care time from adult children				0.0043**	0.0046**
				(0.0020)	(0.0020)
Family Doctor Services					−0.0385
					(0.0301)
Medical service satisfaction					0.0497***
					(0.0057)
Constants	1.2423***	3.1831***	3.2178***	3.1586***	3.0026***
	(0.1345)	(0.1460)	(0.1453)	(0.1462)	(0.1462)
*N*	9921	9921	9921	9921	9921
Adjusted R2	0.0232	0.1825	0.1897	0.1909	0.1974
F	234.7234	366.3933	335.1594	236.8134	207.0551

Note: *, ** and *** indicate significance at the 10%, 5% and 1% statistical levels. Standard errors in parentheses.

### 3.2 Heterogeneity analysis

First, we used T-test to analyze the differences of NHEs in different subgroups of gender, age, household registration and location. The results (Table 3) showed that there were significant differences among the four subgroups of variables. Then, we ran OLS regressions based on different subgroups of gender, age, household registration and location to examine the heterogeneous impact of NHEs on the physical health of the elderly.

**Table 3:** T-test of NHEs by gender, age, household registration and location.

	Group	Obs	Mean	Std. err.	*t*-test
gender	Male	4836	3.4376	0.0091	Pr(|T| > |t|) = 0.0000
	Female	5085	3.1512	0.0095	
age	60-74: the young old	7748	3.3859	0.0071	Pr(|T| > |t|) = 0.0000
	75-89: the old old	2173	2.9515	0.0158	
household registration	Agricultural	6887	3.2564	0.0083	Pr(|T| > |t|) = 0.0000
	Non-agricultural	3034	3.3688	0.0114	
location	Rural Village	4040	3.3717	0.0100	Pr(|T| > |t|) = 0.0000
	Urban Community	5881	3.2352	0.0090	

The regression results in Table 4 show that the NHEs impact the elderly differently depending on gender. The NHEs impact both male and female elderly people positively; in the male subgroup (B = 0.4098, p < 0.01) and in the female subgroup (B = 0.5237, p < 0.01). The World Trade Organization divides the elderly into the young old (60–74 years), the old old (75–89 years), and the longevous (90 years and above). Since the longevous only represented 85 participants (0.86%), we combined this data with that of the old old. The NHEs had a positive impact on physical health in both subgroups: the young old (B = 0.4929, p < 0.01) and the old old/longevous (B = 0.4245, p < 0.01).China has been implementing a Household Registration System, so we divided the sample into agricultural and non-agricultural subgroups and examined the effect of the NHEs on the physical health of the elderly in each subgroup (see Table 4). The NHEs had a positive effect on physical health in both subgroups; the agricultural (B = 0. 5676, p < 0.01) and the non-agricultural (B = 0.2668, p < 0.01). Considering the large-scale population migration that has occurred in China over the past few decades, we also examined the NHEs with regard to the elderly who lived in rural villages or urban communities. In the urban community subgroup, the NHEs (B = 0.2984, p < 0.01) had a positive impact on the physical health of the elderly. In the urban community subgroup, the NHEs (B = 0.2984, p < 0.01) also had a positive impact on the physical health of the elderly.

**Table 4:** Heterogeneity analysis of NHEs by gender, age, household registration and location.

	Male	Female	60-74: the young old	75-89: the old old	Agricultural	Non-agricultural	Rural Village	Urban Community
NHEs	0.410***	0.524***	0.493***	0.425***	0.568***	0.267***	0.552***	0.298***
	−0.051	−0.052	−0.040	−0.092	−0.046	−0.061	−0.049	−0.057
Control variables	YES	YES	YES	YES	YES	YES	YES	YES
*N*	4836	5085	7748	2173	6887	3034	5881	4040
Adjusted R2	0.151	0.170	0.110	0.112	0.195	0.190	0.185	0.201

Note: *, ** and *** indicate significance at the 10%, 5% and 1% statistical levels. YES indicates the control variable within the model.

### 3.3 Robustness tests

(1) Endogeneity problem. Table 5 reports the results of the two-stage least squares estimation based on the instrumental variables method. The results of the one-stage regression in column (1) show that fitness exercise of other elderly people (B = 0.4601, p < 0.01) and leisure activity of other elderly people (B = 0.2129, p < 0.01) both had a significant impact on NHEs, indicating that the two instrumental variables selected for this study satisfied the correlation hypothesis. We also used various statistical tests to evaluate the instrumental variables. The p-value of the Sargan statistic was 0.7735 greater than 0.1, proving the null hypothesis that the instrumental variables were exogenous. This means that they were qualified and independent of the disturbance term. The Kleibergen-Paap rk LM statistic was 737.218, which passed the statistical test at the 1% significance level, thus disproving the null hypothesis that the instrumental variable was unidentifiable. The Kleibergen-Paap rk Wald F statistic was 466.011, and the Cragg-Donald Wald F statistic was 476.487, both of which were greater than the critical value of 19.93 for the Stock and Yogo (2005) test at the 10% level, thus rejecting the instrumental variable is the assumption of weak identification (Kleibergen & Paap, 2006; Stock & Yogo, 2005). The Anderson-Rubin Wald statistic of 17.06 disproved the null hypothesis that the sum of the endogenous regression coefficients was equal to zero at the 1% level, indicating a strong correlation between the instrumental and endogenous variables. Based on the four tests reported in Table 5, we were able to confidently infer that the instrumental variables used in the study were appropriate. The results of the second stage estimation in column (2) showed an estimated coefficient of 0.5018 for the NHEs, which passed the test at the 1% level of significance.
(2) Replacing the independent variable. Neighborhood health in the previous study was assessed at the community level, and here we used the county-level NHEs instead. Due to privacy concerns, CHARLS 2018 did not provide county codes. Fortunately, the actual survey of CHARLS 2018 was conducted in 150 counties in 126 cities, so city-level variables could be used instead of county-level variables. We calculated the county-level NHEs (B = 0.8254, p < 0.01) using Eq. (1) and repeated the calculation as an OLS regression. Column 1 of Table 6 shows that the county-level NHEs (B = 0.8254, p < 0.01) had a positive impact on the physical health of the elderly.
(3) Replacement of dependent variables. When using self-rated health to replace physical health, and switching to O-logit regression, the results showed that the NHEs (B = 0.3758, p < 0.01) had a significant positive impact on self-rated health (Table 6, column 2). However, when using 3-year self-rated health change to replace physical health, and also switching to O-logit regression, we found that the NHEs (B = 0.1257, p < 0.01) also had a significant positive impact on the 3-year self-rated health change (Table 6, column 3).
(4) Different subgroup tests. Based on whether participants had smoked in the past, we divided them into two subgroups, smoked (288 subsamples) and never smoked (4114 subsamples), to test the impact of the NHEs on each category. The results showed that while the health of 288 participants who had smoked in the past was affected by the NHEs (B = 0.5246, p < 0.01) (Table 6, column 4), the health of 4114 participants who had never smoked was even more strongly affected (B = 0.5439, p < 0.01) (Table 6, column 5).

### 3.4 KHB mediation effect analysis

Table 7 presents the proportion of each mediating variable that could be explained separately, and the proportion explained in the full model. Adding social connection to the mediation model alone, the total effect (B = 0.6055), direct effect (B = 0.5455), and indirect effect (B = 0.0601) were all significant (p < 0.01). Adding social relationships to the mediation model alone, the total effect (B = 0.6055), direct effect (B = 0.5756), and indirect effect (B = 0.0299) were all significant (p < 0.01). Social connection and social relationships separately explained 9.92% and 4.94% of the NHEs. When adding both factors into the mediation model, the total effect (B = 0.6056), direct effect (B = 0.5250), and indirect effect (B = 0.0800) of NHEs were significant (p < 0.01), and the total effect was 1.1526 times larger than the direct effect, and 13.24% of the total effect came from these two factors.

**Table 5:** NHEs on the physical health of the elderly (IV-2SLS).

	First-stage regressions	IV (2SLS) estimation
	NHEs	Physical Health
NHEs		0.5018***
		(0.1210)
NFEs (Neighborhood fitness exercises)	0.4601***	
	(0.0332)	
NLAs (Neighborhood leisure activities)	0.2129***	
	(0.0098)	
Control variables	YES	YES
Sargan statistic	0.083(p = 0.7735)	
Under-identification test		
Kleibergen-Paap rk LM statistic	737.218***	
Weak identification test		
Kleibergen-Paap rk Wald F statistic	466.011***	
Cragg-Donald F statistic	476.487***[19.93]	
Weak-instrument robust inference		
Anderson-Rubin Wald	17.06***	
*N*	9921	9921
Centered R2		0.1983

Note: *, ** and *** indicate significance at the 10%, 5% and 1% statistical levels. Standard errors in parentheses. YES indicates the control variable within the model. [19.93] indicates the critical value of the Stock and Yogo (2005) test at the 10% level.

**Table 6:** Robustness tests of NHEs.

	Physical Health	Self-rated health (O-logit)	3-year self-rated Health Changes (O-logit)	Smoked	Never smoked
County-level NHEs	0.8254***				
	(0.0518)				
NHEs		0.3758***	0.1257***	0.5246***	0.5439***
		(0.0679)	(0.0441)	(0.2012)	(0.0526)
Control variables	YES	YES	YES	YES	YES
*N*	7805	7803	7798	288	4114
Adjusted R2	0.1737	0.0478	0.0159	0.1711	0.1560

Note: *, ** and *** indicate significance at the 10%, 5% and 1% statistical levels. Standard errors in parentheses. YES indicates the control variable within the model.

**Table 7:** Mediation effect analysis of NHEs (KHB).

	Online	lnCG		Online & lnCG
Total effect	0.6055***	0.6055***	Total effect	0.6056***
	(0.0391)	(0.0389)		(0.0387)
Direct effect	0.5455***	0.5756***	Direct effect	0.5250***
	(0.0393)	(0.039)		(0.0390)
Indirecte effect	0.0601***	0.0299***	Indirecte effect	0.0800***
	(0.0072)	(0.0063)		(0.009)
Separate explanations	9.92%	4.94%	Confounding ratio	1.1526
Full model explanation	8.70%	4.54%	Confounding percentage	13.24%

Note: *, ** and *** indicate significance at the 10%, 5% and 1% statistical levels. Standard errors in parentheses.

## 4 Discussion

### 4.1 Key findings

We found that the NHEs (p < 0.01) consistently had a positive impact on the physical health of the elderly, after controlling for individual basic status, economic status, family care status, and public medical policy variables. Younger elderly people were healthier, and the eight variables of gender, marital status, education, lnTPI, proportion of sons, lnFSC, care time from adult children, family doctor services, and medical service satisfaction were significant and positively impacted the health level of the elderly; the effects of these variables were consistent with expectations (Feng et al., 2019). Household registration was non-significant in all models. A possible reason for this is that household registration indicates whether the elderly live in urban communities or rural villages. There are considerable differences between these residents in China in terms of education, income, and access to health care resources, so some of the effects of household registration were dispersed to other factors such as education, economy, and health care resources. Like Long-Term Care Insurance (LTCI), Family Doctor Services was piloted in China in June 2016, and the policy effects were not fully reflected by the short implementation time and a low number of participants in this program in 2018 (Chen & Xu, 2020). Our data show that only 4.43% of the elderly (440 cases) participated in this program, which may explain the non-significance of Family Doctor Services. It is noteworthy that the contracting rate of family doctor and key populations exceeded 60% in 2021, and the coverage of family doctor contracting services is projected to reach more than 75% by 2035, so more research on Family Doctor Services is needed in the future.

The study showed that the NHEs differed across groups. First, they were less impactful in the male subgroup than the female one, by approximately 27.79%. Given that the neighborhood effect relies heavily on neighborhood networks and relationships, we speculate that there are two reasons for this difference. One is that females are generally healthier than males of the same age, so they have more physical strength and energy to participate in neighborhood activities. The second is that in the traditional Chinese household division of labor, women take on more tasks such as shopping and caring for grandchildren, so they have more opportunities to participate in neighborhood activities (Chrisinger et al., 2022). Second, the NHEs also showed some differences in different age subgroups, with the young old subgroup exhibiting a greater impact of NHEs on physical health. Specifically, for each 1-unit increase in the NHEs, the young old's physical health increased by 0.4929 units, while the old old's physical health increased by only 0.4245 units, a difference of 16.11%. The impact of the NHEs on the physical health of the elderly shows a weakening trend with increasing age. This may be because older seniors are also less healthy and participating in neighborhood interactions and communication is correspondingly reduced; thus, the health benefits obtained are also reduced. Finally, we tested for differences in NHEs across household subgroups (Han & Chung, 2022). The NHEs had a much larger impact on the physical health of agricultural elderly individuals than non-agricultural ones. For each 1-unit increase in the NHEs, the physical health of the agricultural elderly increased by 0.5676 units, while the physical health of their non-agricultural counterparts increased by only 0.2668 units, a difference of 197%. In the rural village subgroup, the coefficient of the NHEs on physical health of the elderly was 0.5519, while in the urban community subgroup, this coefficient was only 0.2984. This is generally consistent with the conclusion resulting from differences in household registration. We infer two causes that may account for this. First, since ancient times, Chinese rural villages have been societies of acquaintances, which Dr. Fei Xiaotong called “the Pattern of Difference Sequence”. The difference is that urban communities in modern China are societies of strangers; therefore, the NHEs are more influential in rural villages (Fei et al., 1992). Second, over the years, rural village education has lagged behind urban community education, so the elderly in rural villages are more likely to accept and put into action the health knowledge acquired from their neighbors (Han & Chung, 2022).

### 4.2 What this study adds

Although in the OLS baseline model and heterogeneity analysis, we reached the conclusion that the NHEs have a positive impact on the physical health of the elderly, these are based on the same model and variables. Therefore, the reliability of the conclusions still called for more testing. We used four approaches to test reliability. One was to construct two instrumental variables: NFEs and NLAs (Carta et al., 2021). Then, we used the IV-2SLS to solve the problem that physical health and NHEs of the elderly may be mutually causally affected. These two lifestyle factors impact the health of the elderly who are directly participating and have little effect on those who are not. By comparing the OLS regression results with the IV-2SLS regression results, we found that the NHEs coefficient reached a significant level of 1% before and after the elimination of the endogeneity problem. However, after eliminating the endogeneity problem, the NHEs coefficient increased from 0.4689 to 0.5018. This indicates that endogeneity caused bias to the estimation results, while adopting the instrumental variables significantly improved the baseline regression results. This once again validated the positive impact of the NHEs on the physical health of the elderly. Second, the independent variable was replaced. A higher county-level NHEs were used to replace the community-level NHEs. County-level administrative divisions have been used for many years in China, and nowadays China also uses county-level government as the basic unit to formulate public policies. Therefore, not only does a community share the same history, customs, dialects, and public policies, but these will lead to more networks and relationships between the elderly within the county. Therefore, county-level NHEs and community-level NHEs are correlated, which confirms that higher-level NHEs also positively and significantly impact the physical health of the elderly. The third approach was to replace the dependent variables with self-rated health and 3-year health change. Self-rated health is generally considered to be a combined assessment of self-physical health and mental health, so using this variable can compensate to some extent for the lack of adoption of physical health (Yang et al., 2018; Zhang & Wu, 2017). CHARLS 2018 asked: Would you say your health is very good, good, fair, poor, or very poor? We used self-rated health as a replacement for physical health and switched the model, and the results showed that NHEs also had a positive impact on self-rated health in the elderly. In addition, physical health obtained from cross-sectional data measures ignores the impact of health history and current physical health instability in stages. We used the 3-year self-rated health change to mitigate these two issues. CHARLS 2018 asked: Compared with your health when we talked with you last time (3 years ago), would you say that your health is better now, about the same, or worse? After constructing the health change variable and switching to the O-logit model, we found that the NHEs had a similarly significant positive impact on the 3-year self-rated health change of the elderly. Finally, we performed different sample tests, especially to determine the robustness of the model in small samples. Smoking is generally considered to have an impact on health (Huang et al., 2021). In this study, the elderly were divided into two groups based on whether they had smoked in the past or not. In a large sample of 4114 (never smoked), the NHEs had a positive impact on the physical health of elderly persons. Meanwhile, in the small sample of 288 (smoked), the results still supported the positive impact of the NHEs on physical health. Therefore, the impact of NHEs is always robust, regardless of smoking habits, in both large and small samples.

### 4.3 Strengths and limitations

We have two Strengths. First, we constructed a comprehensive research and analysis framework integrating OLS regression, heterogeneity analysis, IV-2SLS, robustness tests, and KHB mediated effect analysis to thoroughly examine the impact of NHEs on the physical health of the elderly in China. Second, we identified the mechanism of the impact of the NHEs on the health of the elderly in terms of social networks and social relationships. The results showed that the mediating effect of social connection and social relationships was significant, and 13.24% of the NHEs originated from the proxy indicators of these factors, internet use and cash gifts. This means that changes in social networks (“online”) have not only triggered a paradigm change in accessing and sharing health information, but also promoted the diffusion of NHEs and enhanced their function, all of which contribute to improving the health of the elderly (Booth et al., 2018; Huang et al., 2021). Social relationships have a significant indirect effect on enhancing the physical health of the elderly. Therefore, establishing a safe community environment and friendly neighborhood relations not only improves the strength of neighborhood social relations, but benefits the elderly(Ma et al., 2020; Wang et al., 2019).

However, two limitations of this study must be noted. First, it used cross-sectional physical health data. Although robustness tests were conducted using self-rated and 3-year self-rated health change variables, they cannot fully compensate for the bias caused by the absence of mental health and health history. Second, this study tested the transmission mechanism of the NHEs on the physical health of the elderly, but the mediating effect was small, suggesting that there may be other more important transmission mechanisms.

## 5 Conclusions

Our study used data from the China Health and Retirement Longitudinal Study (CHARLS) 2018, focusing on the impact of NHEs on the physical health of the elderly. We obtained four main conclusions. First, there were heterogeneous differences in NHEs, with higher NHEs for females, the young old, and in agricultural and rural village subgroups. Second, after controlling for endogeneity problems, OLS underestimated the impact of the NHEs compared to IV-2SLS. Third, using the city-level NHEs, self-rated health, and 3-year self-rated health change replacement variables, both in the smoked (small sample) and never smoked (large sample) subgroups, the influence of NHEs on the physical health of the elderly was robust in the study. Finally, social networks and social relationships are important transmission mechanisms of the NHEs with regard to the physical health of the elderly.

This study provides a new viewpoint for understanding the increasing number of community fitness facilities and smart community transformations in China from the perspective of NHEs. Combining the conclusions of this study, we propose three suggestions. First, we suggest strengthening community infrastructures such as roads, networks, parks, and activity centers, as well as organizing various fitness exercises and leisure activities for residents, in order to break the shackles of the region's inherent social networks. Second, to encourage more interaction and to share among residents, we advocate creating a strong culture of mutual assistance among neighbors and guiding residents to build warm and harmonious neighbor relationships. Third, communities should do what they can to help the elderly adapt to technology, and strengthen “smart age” software and hardware to accommodate aging. For example, we should optimize audio and video interaction, expand online health care services, and increase remote leisure programs and network resources to promote the health of the elderly. Four, future studies could use panel studies or composite health indicators to explore the impact of NHEs on the health of the elderly, while the transmission mechanisms of NHEs need to be explored more.

## Ethical statement

Ethical approval for all the CHARLS waves was granted from the Institutional Review Board at Peking University. The IRB approval number for the main household survey, including anthropometrics, is IRB00001052-11015; the IRB approval number for biomarker collection, was IRB00001052-11014.

During the fieldwork, each respondent who agreed to participate in the survey was asked to sign two copies of the informed consent, and one copy was kept in the CHARLS office, which was also scanned and saved in PDF format. Four separate consents were obtained: one for the main fieldwork, one for the non-blood biomarkers and one for the taking of the blood samples, and another for storage of blood for future analyses.

## Author statement

Author 1 (First Author): Conceptualization, Methodology, Software, Writing - Original Draft, Writing - Review & Editing.

Author 2 (Corresponding Author): Data, Methodology, Writing - Original Draft, Writing - Review & Editing.

Author 3: Methodology, Writing - Review & Editing.

## Data Availability

The authors do not have permission to share data.

## Footnotes

1 Note: The Chinese population in this study refers to the population of mainland China, excluding residents of Hong Kong, Macao, Taiwan, and foreigners living in mainland China.
